# Suppurative Meningoencephalitis by *Pseudomonas aeruginosa* from Direct Extension of Chronic Otitis in a Gir Cow

**DOI:** 10.3390/vetsci10060398

**Published:** 2023-06-16

**Authors:** Antonio Carlos Lopes Câmara, Davi Emanuel Ribeiro de Sousa, Isabel Luana de Mâcedo, Karoline Lacerda Soares, José Renato Junqueira Borges, Carlos Frederico Martins, Adriano Queiroz de Mesquita, Valéria Dutra, Márcio Botelho de Castro

**Affiliations:** 1 Large Animal Veterinary Teaching Hospital, College of Agronomy and Veterinary Medicine, University of Brasília, Brasilia 70910-900, DF, Brazil; jrborges@unb.br; 2 Veterinary Pathology Laboratory, College of Agronomy and Veterinary Medicine, University of Brasília, Brasilia 70910-900, DF, Brazil; daviers@hotmail.com (D.E.R.d.S.); karoline_lacerda@hotmail.com (I.L.d.M.); isabeluanamacedo@gmail.com (K.L.S.); mbcastro@unb.br (M.B.d.C.); 3 Centro de Tecnologia para Raças Zebuínas Leiteiras, Embrapa Cerrados, Brasilia 70770-901, DF, Brazil; carlos.martins@embrapa.br (C.F.M.); adriano.mesquita@embrapa.br (A.Q.d.M.); 4 Laboratory of Veterinary Molecular Biology, Veterinary Hospital, Federal University of Mato Grosso, Cuiabá 78060-900, MT, Brazil; valdutra@ufmt.br

**Keywords:** cattle, central nervous system, cerebrospinal fluid, suppurative intracranial infections

## Abstract

**Simple Summary:**

This paper reports a case of suppurative meningoencephalitis by *Pseudomonas aeruginosa* from the direct extension of chronic otitis in a Gir cow. The direct extension of otitis media/interna results in secondary pyogranulomatous intracranial lesions occasionally. Clinical signs included forebrain, cerebellum, cranial nerve VII, VIII, and XII impairment. Pure cultures of *P. aeruginosa* were obtained and identified in the suppurative central nervous system (CNS) lesions, meninges, and inner ear samples. The paucity of case reports of middle or inner ear infections evolving to suppurative processes in the CNS of adult cattle suggests underreporting or lack of diagnosis.

**Abstract:**

This paper aims to report clinical, laboratory and pathological features in a case of suppurative meningoencephalitis by *P. aeruginosa* from the direct extension of chronic otitis in a Gir cow. The cow was recumbent during physical examination, and neurological examination revealed depression, absence of left eyelid and auricular motor reflex, and hypotonic tongue. Hematology revealed hemoconcentration, leukocytosis by neutrophilia, and hyperfibrinogenemia. Cerebrospinal fluid was slightly turbid, and presented polymorphonuclear pleocytosis, and hyperproteinorrachia. Grossly, the skull floor showed a purulent green–yellow exudate that drained from the left inner ear to the cisterna magna. There was diffuse congestion of the telencephalon, and meninges showed severe hyperemia, moderate thickening, and opacity with the deposition of fibrinosuppurative material ventrally, extending to the cerebellum and brainstem. The left cerebellar hemisphere showed an approximately 1.5 cm in diameter liquefaction area surrounded by a hemorrhagic halo. Histologically, cerebellar, mesencephalic, thalamic, and brain base meninges were intensely thickened and showed severe suppurative inflammation and fibrin deposition. Small multifocal suppurative areas were observed in the cerebellum and brainstem, characterized by a necrotic core, a number of neutrophils, and Gram-negative intralesional bacillary myriads. Pure cultures of *P. aeruginosa* were obtained and identified in the suppurative CNS lesions, meninges, and inner ear samples. This report highlights an uncommon clinical evolution of secondary *P. aeruginosa* suppurative meningoencephalitis, probably triggered by recurrent parasitic otitis in an adult Gir cow. Veterinarians, practitioners, and farmers must be aware of the risk of CNS infections after unresolved media and inner otitis, especially in cattle breeds more prone to developing parasitic otitis, such as the Gir and Indubrasil breeds.

## 1. Introduction

The central nervous system (CNS) may be affected by infectious agents through four paths: from hematogenous or lymphatic dissemination deriving from distant sites; direct penetrating lesions; through an adjacent suppurative lesion; or centripetal ascending infection through peripheral nerves. Direct extension of necro-suppurative processes involving other structures of the skull, such as head trauma, dehorning lesions, and otitis media/interna, result in secondary pyogranulomatous intracranial lesions occasionally [1,2,3]. Despite this, the paucity of case reports of middle or inner ear infections evolving to suppurative processes in the CNS of adult cattle suggests underreporting or lack of diagnosis.

*Pseudomonas aeruginosa* is an aerobic Gram-negative bacillus and an opportunistic pathogen that has been implicated in different opportunistic infections and nosocomial outbreaks in humans [4,5,6]. *P. aeruginosa* is a significant human pathogen and also affects animals worldwide [5]. Recent reports associated with *P. aeruginosa* infection in cattle are restricted to otitis [7,8], esophageal and glossal ulceration [5] in calves, and mastitis in cows [9,10]. The only description of *P. aeruginosa* meningoencephalitis in adult cattle is secondary from frontal sinusitis in a beef bull, but this study lacks a detailed pathological analysis [11]. Therefore, this report describes clinical, laboratory, and pathological features of a case of suppurative meningoencephalitis by *P. aeruginosa* from direct extension of chronic otitis in a Gir cow.

## 2. Case Presentation

A 12-year-old and 400 kg cow from a high genetic value Gir Mocho herd was the case subject. The cattle herd was raised extensively at a farm in Brasília-DF, Midwestern Brazil, and they were vaccinated for foot-and-mouth disease and rabies. Several cases of otitis by *Rhabditis* sp. were observed in the Gir Mocho herd, proven by the combined clinical signs and visualization of the nematode after ear lavage. Initially, this Gir cow presented bloody discharge on the left ear. Due to the herd’s parasitic otitis background, the practitioner recommended cleaning the ear canal followed by topical treatment with a mixture solution (trichlorfon, oxytetracycline, and dimethyl sulfoxide) for five days. A slight improvement in the clinical condition was observed, but seven days after the end of the treatment, the cow presented ataxia and a left-sided head tilt. Topical treatment was repeated in the ears in association with a single intramuscular injection of long-acting enrofloxacin (5 mg·kg^−1^), but there was no clinical improvement. Therefore, the Gir cow was referred for hospital care after 14 days of clinical evolution.

At hospital admission, anamnesis and systematic clinical examination [12] were performed. Physical examination revealed lateral recumbency and an inability to achieve sternal recumbence, even if assisted. The Gir cow presented a regular body condition score (5/10 score), dehydration (assessed by skin turgor), hypothermia (35 °C, 95 °F), and ruminal and intestinal hypomotility. Neurological examinations revealed depression, absence of left eyelid and auricular motor reflex, and hypotonic tongue. During manipulation, the cow presented sporadic pedaling movements. We could not assess gait and proprioception due to the cow’s inability to stand. Discharge from the ears was not noticed during the admission evaluation.

Blood samples were collected through jugular venipuncture for hematology (complete blood count and fibrinogen determination), and serum biochemistry profiling (aspartate aminotransferase [AST] and gamma-glutamyl [GGT] transferase activities, total protein, albumin, urea, and creatinine levels). Hematology revealed hemoconcentration (hematocrit: 42%; hemoglobin: 14.7 g/dL; red blood cells: 10.17 × 10^3^/μL), leukocytosis (17.6 × 10^3^/μL; reference range: 4–12 × 10^3^ leukocytes/μL) by neutrophilia (12.4 × 10^3^/μL; reference range: 0.6–4.0 × 10^3^ neutrophils/μL) and hyperfibrinogenemia (700 mg/dL; reference range: 200–600 mg/dL) [13]. Biochemical abnormalities included hypoalbuminemia (1.7 g/dL; reference range: 3.03–3.55 mg/dL), increased AST (246 U/L; reference range: 20–34 U/L), and GGT (22 U/L; reference range: 6.1–17.4 U/L) activities [14]. Cerebrospinal fluid (CSF) was collected from the atlanto-occipital space for biochemical and cytological analysis [15], revealing slight turbidity, polymorphonuclear (77% neutrophils, 19% lymphocytes, 3% monocytes, and 1% eosinophils) pleocytosis (1278 nucleated cells/µL, normal range: 0–7 cells/µL), and hyperproteinorrachia (1700 mg/dL, normal range: 24–40 mg/dL) [15].

**Figure 1:**
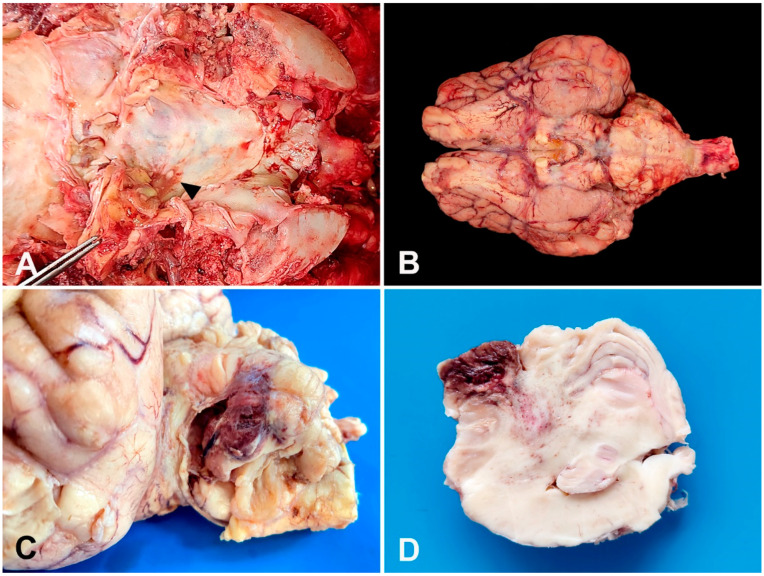
A 12-year-old Gir cow. Gross findings. (**A**) Purulent green–yellow exudate within the left inner ear that drains to the skull floor (arrow) and cisterna magna. (**B**) Brain’s ventral surface. Severe meningeal hyperemia, thickening, and opacity with deposition of fibrinosuppurative exudate. (**C**) Left cerebellar hemisphere. Formalin-fixed sample. Suppurative area of 1.5 cm in diameter with central malacia surrounded by a hemorrhagic halo. (**D**) Cerebellum cut surface. Formalin-fixed sample. Cerebellar asymmetry with malacia and hemorrhage extending from the cerebellar vermis to the cerebellar peduncle.

A presumptive diagnosis of meningoencephalitis was proposed. The Gir cow was humanely euthanized (0.1 mg·kg^−1^ of xylazine and thiopental overdose intravenously followed by an intrathecal injection of 100 mL lidocaine) [16] after the owner’s consent due to poor prognosis, and a necropsy was performed. Grossly, the brain’s ventral surface showed a purulent green–yellow exudate deposition that drained from the left inner ear to the skull floor and cisterna magna (Figure 1A). There was diffuse congestion of the telencephalon, and the meninges showed severe hyperemia, moderate thickening, and opacity with the deposition of fibrinosuppurative material ventrally (Figure 1B). The most affected regions included the optic chiasm and piriform lobe extending to the brainstem and cerebellum. The left cerebellar hemisphere showed an approximately 1.5 cm in diameter suppurative area with a central liquefaction surrounded by a hemorrhagic halo (Figure 1C). The cut surface of the cerebellum and brainstem showed asymmetry with multifocal malacia areas filled by a suppurative content and hemorrhage extending from the left cerebellar hemisphere and vermis to the cerebellar peduncle (Figure 1D). No gross lesions were observed on other CNS regions.

**Figure 2:**
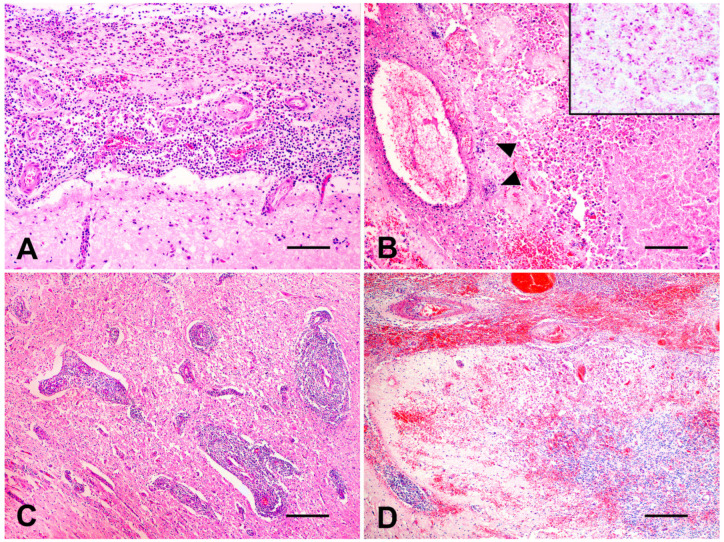
A 12-year-old Gir cow. Histological findings. (**A**) Brain’s ventral surface. Severe suppurative meningitis (H&E, bar = 100 µm). (**B**) Cerebellum. Multifocal areas of necrosis and marked suppurative inflammation with multifocal bacterial myriads (head arrows) (H&E, bar = 100 µm). Inset: close view of Gram-negative intralesional bacteria (Gram stain). (**C**) Brain’s ventral surface. Numerous mononuclear perivascular cuffs (H&E, bar= 250 µm). (**D**) Cerebellum. Severe multifocal to coalescing hemorrhage affecting meninges and parenchyma (H&E, bar = 250 µm).

Organs and tissues collected were fixed in 10% buffered formalin (pH 7.0), routinely processed for histopathology, and histological sections were stained with hematoxylin and eosin (H&E) (all samples) and Gram stain (cerebellum and brainstem samples). Microscopically, cerebellar, mesencephalic, thalamic, and brain base meninges were intensely thickened and showed severe suppurative inflammation (Figure 2A) and fibrin deposition. Small multifocal suppurative areas were observed in the cerebellum and brainstem, characterized by a necrotic core, a number of neutrophils, and Gram-negative intralesional bacillary myriads (Figure 2B). Intact and degenerated neutrophils, Gitter cells, fibrin, axonal spheroids, neuropil vacuolation, mononuclear perivascular cuffs (Figure 2C), hyperplasia of endothelial vascular cells, and hemorrhage (Figure 2D) were observed surrounding severely affected areas. A moderate lymphohistiocytic inflammatory infiltrate with scattered neutrophils and eosinophils and mild fibrosis was underlying the left auditory tube epithelium.

Fresh samples were collected from the suppurative CNS lesions, meninges, and inner ear, and seeded on 8% ovine blood agar medium (Sigma-Aldrich, Darmstadt, Germany) and MacConkey agar (Neogen Corporation, São Paulo, Brazil) medium at 37 °C in aerobiosis. Pure cultures of *P. aeruginosa* were obtained and identified in the brain’s suppurative areas, meninges, and inner ear samples. Isolates were identified based on the standard bacteriological approaches, such as colony morphology, pyocyanin pigment production, Gram staining, growth at 44 °C, and the following biochemical tests: oxidase, catalase, nitrate reduction, indole, methyl red, Voges-Proskauer, citrate utilization, and glucose fermentation [17].

## 3. Discussion

Infections in the nervous system are among the most lethal diseases and generally have a substantial economic impact on cattle herds. These infections affecting the cerebrum or forebrain (cerebral hemispheres, thalamus, and hypothalamus), such as in bacterial meningitis, are considered infrequent in adult ruminants [1,2,3,18], but are more commonly seen in young animals [7,8].

CNS infections may cause a number of clinical signs depending on the anatomical location and severity of the lesions [1,3]. Initial clinical signs observed in the Gir cow suggest the involvement of different parts of the CNS, including the forebrain (altered mentation), cerebellum (ataxia), and vestibulum (head tilt to the side of the lesion). Additionally, cranial nerve VII (inability to blink), VIII (head tilt), and XII (hypotonic tongue) dysfunction detected on admission also suggest brainstem impairment. The space-occupying nature of the suppurative CNS lesions, in association with diffuse forebrain involvement caused by bacterial meningoencephalitis, can explain the number of neurological signs observed in the Gir cow [1,2,18].

Cattle with long and cannulated ears, such as the Gir and Indubrasil breeds, are more prone to developing parasitic otitis by nematodes of the genus Rhabditis (Rhabditidae) [19]. Although the physical evaluation has not verified the parasitic otitis in the Gir cow, the herd has a proven history of Rhabditis-related otitis. Bovine auricular parasitosis has a complex dynamic and low post-treatment cure rate, predisposing the recurrence and secondary opportunist bacterial infections [19,20]. Recently, the use of 1% dimethyl sulfoxide and mineral oil in conjunction with nematophagous fungi presented promising results to control *Rhabditis* spp., enhancing the parasitic otitis treatment in the field [20].

In addition to the clinical signs, hematology abnormalities detected in the cow, such as severe neutrophilic leukocytosis and hyperfibrinogenemia, might be related to the acute bacterial inflammatory process in the CNS [13]. Additionally, CSF analysis showed polymorphonuclear (mainly neutrophilic) pleocytosis and a marked hyperproteinorrachia, supporting the diagnosis of CNS bacterial infection with a blood–brain barrier disruption [1,2,15]. Usually, the aforementioned CSF changes are associated with suppurative CNS processes, such as brain or spinal cord abscesses and septic meningoencephalomyelitis [1,2,12].

Bacterial encephalitis, by direct extension, has been associated with infections adjacent to the CNS, mainly affecting the pituitary fossa, paranasal sinuses, cribriform plate, and inner ear [21,22]. The cribriform plate and the inner ear are considered the most common pathways for bacterial infections in the CNS due to the direct anatomical communication and the significant vascularization of these structures [21]. Additionally, meningitis can be caused by bacterial migration throughout the eustachian tube in cattle [8]. In addition to the clinical and laboratory findings, gross and histological findings observed in the CNS support the hypothesis that parasitic otitis is followed by secondary bacterial infection extended to the CNS, promoting suppurative inflammation in the Gir cow.

One of the most relevant findings in this report was the identification of *P. aeruginosa* as the etiological agent of suppurative CNS lesions in the cow. Although infectious causes of cerebral disease in adult ruminants are infrequent [18], *Trueperella pyogenes* is considered the the most common bacteria in chronic suppurative lesions of the brain [1,3]. However, in calves, *Escherichia coli* is generally the most common pathogen associated with meningitis, but other bacteria such as *Salmonella*, *Campylobacter*, *Klebsiella*, and different *Staphylococcus* species have also been reported [2,23]. *P. aeruginosa* is a significant cause of nosocomial infections worldwide and has been associated with media and inner otitis in humans [4,6], and sporadically in calves [7] and sheep [24]. In ruminants, *P. aeruginosa* is frequently reported as a relevant environmental and opportunistic pathogen causing mastitis [9,10], lymphadenitis [25], hepatic abscesses [26], rhinitis, and dermatitis [24].

Malacia with loss of typical CNS architecture and suppurative inflammation with intralesional bacteria observed in cattle possibly resulted from the action of *P. aeruginosa* virulence factors such as flagellin, type IV pilin, phospholipase C, protease IV, alkaline protease (*apr*A), staphylolysin, elastase, exoenzymes (S, T, and U) and exotoxins [25,27]. Phospholipase C encoded by the *plc*H gene of *P. aeruginosa*, together with the *alg*D gene, promotes alginate biosynthesis, which protects the bacteria from antibiotics and the host’s immune response, conferring a multi-drug-resistant feature [27]. *P. aeruginosa* infections trigger tissue injury through neutrophilic inflammation mediated mainly by releasing IL-1β and IL-8. The activation of these interleukins depends on intracellular recognition receptors called inflammasomes formed from recognizing molecular patterns present in bacteria such as flagellin, exolysin, and exotoxin A lipopolysaccharide [28]. Peak expression of IL-6, IL-11, transforming growth factor (TGF)-β1, and tumor necrosis factor (TNF)-α from one to three days after infection are also related to tissue damage [29]. Another form of bacterial survival in tissues is related to virulence phenotypes, such as *apr*A, *exo*U, and *exo*S that decrease the activation of Toll-like receptor 5 (TLR-5) linked to the complement system, reducing the action of the immune system against the pathogen [29].

Bacterial encephalitis is a difficult-to-treat and fatal condition in ruminants [1,3,7]. Thickened opaque leptomeninges with fibrin deposition associated with necrosis and hemorrhage of the neuroparenchyma are lesions that are also observed in meningoencephalitis by *E. coli*, *Campylobacter*, *Histophilus somni*, *T. pyogenes*, *K. pneumoniae*, *Chlamydia pecorum* and *Naegleria fowleri* in cattle [1,2,3,23,30,31,32]. Septicemic bacterial leptomeningitis and meningoencephalitis have been observed in calves in the neonatal period caused by *Streptococcus* sp., *Salmonella* sp., *Mycoplasma* sp., *Chromobacterium violaceum*, *Pasteurella* sp. and *T. pyogenes* [1,2,3,8]. Neurolisteriosis is also a striking bacterial infection in adult cattle but with lesions restricted to the brainstem [33]. We reiterate that treatment of bacterial meningoencephalitis is effective only when instituted early in the disease course. If neurologic signs have been present for several days or if recumbency or paralysis has developed, treatment is usually unrewarding [7]. Additionally, early identification of opportunistic *P. aeruginosa* infections is essential to select the correct therapeutics and to set up a prognosis since antibiotic treatment is usually ineffective due to widespread bacterial resistance to many first-line antibiotics [25].

## 4. Conclusions

This report highlights an uncommon clinical evolution of secondary *P. aeruginosa* suppurative meningoencephalitis, probably triggered by recurrent parasitic otitis in an adult Gir cow. Veterinarians, practitioners, and farmers must be aware of the risk of CNS infections after unresolved media and inner otitis, especially in breeds of cattle more prone to develop parasitic otitis, such as the Gir and Indubrasil breeds.

## Data Availability

Not applicable.
